# Production of Workpieces from Martensitic Stainless Steel Using Electron-Beam Surfacing and Investigation of Cutting Forces When Milling Workpieces

**DOI:** 10.3390/ma16134529

**Published:** 2023-06-22

**Authors:** Nikita V. Martyushev, Victor N. Kozlov, Mengxu Qi, Vadim S. Tynchenko, Roman V. Kononenko, Vladimir Yu. Konyukhov, Denis V. Valuev

**Affiliations:** 1 Department of Materials Science, Tomsk Polytechnic University, 634050 Tomsk, Russia; 2 Department of Mechanical Engineering, Tomsk Polytechnic University, 30, Lenin Str., 634050 Tomsk, Russia; kozlov-viktor@bk.ru (V.N.K.); mensyuy1@tpu.ru (M.Q.); 3 Department of Technological Machines and Equipment of Oil and Gas Complex, School of Petroleum and Natural Gas Engineering, Siberian Federal University, 660041 Krasnoyarsk, Russia; vadimond@mail.ru; 4 Information-Control Systems Department, Institute of Computer Science and Telecommunications, Reshetnev Siberian State University of Science and Technology, 660037 Krasnoyarsk, Russia; 5 Digital Materials Science: New Materials and Technologies, Bauman Moscow State Technical University, 105005 Moscow, Russia; 6 Computer Hardware and Software Laboratory, Institute of Information Technologies and Data Analysis, Irkutsk National Research Technical University, 664074 Irkutsk, Russia; 7 Department of Automation and Control, Irkutsk National Research Technical University, 664074 Irkutsk, Russia; 8 Yurga Technological Institute (Branch), Tomsk Polytechnic University, 26, Leningradskaya Street, 652055 Yurga, Russia

**Keywords:** electron-beam surfacing, additive technologies, martensitic stainless steel, cutting forces, machining conditions, milling

## Abstract

The aim of this study was to investigate cutting force when milling 40 × 13 stainless steel samples obtained via electron-beam surfacing. The samples were obtained by surfacing the wire made from the martensitic 40 × 13 stainless steel. The microstructure of the samples and the hardness are discussed in the present study. Emphasis is placed on the study of cutting forces when handling the samples. The structure of the samples obtained by electron-beam surfacing consisted of tempered martensite. The average hardness of the samples was similar to the hardness obtained after quenching and tempering the samples—576 HV for horizontally printed workpieces and 525 HV for vertically printed workpieces. High-speed milling, high-efficiency milling, and conventional milling have been proven to be suitable for handling such workpieces. This study shows that an increase in milling width leads to a gradual decrease in specific cutting force. As the milling depth increases, the specific cutting force decreases intensively at first but then more slowly with time. Machining the workpieces made of the martensitic stainless steel and produced by electron-beam surfacing requires the use of purely carbide mills with a diameter of at least 12 mm. Using a high-speed steel as a tool material results in the rapid failure of the tool. The cutting conditions during the investigation allowed for a decrease in the temperature of the cutting edge, cutting force, and the low-rigid end mill bending. Therefore, this study has made it possible to select modes that allow for a reduction in the vibration of the lathe-fixture-tool-part system.

## 1. Introduction

In recent years, much attention has been paid to the additive technologies involved in wire printing. Such 3D printing has high productivity and a low cost of manufacturing parts. The main disadvantage of such technologies is the poor quality of the formed surface, irregularities, and large roughness of the surface, which requires subsequent machining. When machining an uneven surface, cutting tools vibrate, which accelerates their wear. Due to the peculiarities of 3D printing with wire, the workpiece hardness is significantly higher than that produced by conventional forging. In particular, this concerns martensitic stainless steels. These steels are relatively inexpensive; therefore, they are quite widely used. The good corrosion resistance and high strength of martensitic stainless steels enable the manufacturing of turbine blades, shafts, and surgical instruments. However, during 3D printing with wire, this material significantly changes its properties.

An important factor influencing the formation of the martensitic steel properties when using additive technologies is the layer-wise application of the material. In case of such application, the previous layer undergoes rapid reheating and rapid cooling. The critical cooling rate of the martensitic stainless steel is much lower than the cooling rate of the sample layer when using the additive technologies associated with wire printing. This is relevant for the laser, arc, and electron-beam printing of samples. The authors of [1] have justified this fact in their research. For instance, the critical speed for 40 × 13 stainless steel (0.4% of C; 13% of Cr) is 0.5 K/s.

The cooling rate for the 3D printing methods mentioned above is significantly high; therefore, a martensitic structure will be formed. In this case, heating and cooling cycles initiate processes similar to quenching and tempering, which generates a heterogeneous microstructure. The printed samples will consist of different areas that smoothly pass into one another. These areas will have different phase compositions, different grain sizes, and, as a result, different properties.

The authors of [2,3] studied the mechanical properties and structure of stainless steel samples printed by laser-based powder bed fusion (LPBF). They noted that the properties were different in different directions. Hardness and tensile strength could be changed. The grain size and porosity varied throughout the structure.

The relationship between microstructure and properties was shown by the authors of [4]. This work proves that the properties of printed products can be changed via subsequent heat treatment. However, this additional operation requires additional costs. Similar results are shown in [5], demonstrating that, as a result of the thermal cycling of separate areas during printing, internal stresses may occur in the printed workpiece. The authors of [5] state that a hard-to-treat crust (750 HV) was formed on the surface of the 40 × 13 samples during SLM.

When printing with wire (WAAM), the lower layer is recrystallized when applying a subsequent layer on it. In this case, a structure consisting of elongated ferrite grains and fine-grained needle-like martensite in a matrix in the upper layer is formed. This structure is formed instead of the spatial periodicity of martensitic rails inside equiaxed ferrite grains in the inner deposited coatings. The martensite content gradually increases as it becomes withdrawn from the base metal [6]. Another effect that the authors noted was that a thin layer of metal oxide covered the surface of each layer when manufacturing WAAM stainless steel parts.

In many ways, the formed part parameters will be determined by the printing modes. The process of additive manufacturing using wire is highly dependent on the surfacing conditions. These are parameters such as the substrate temperature, the movement trajectory, etc. [7]. However, despite optimal conditions, structure heterogeneities, surface layer hardening, and other undesirable effects are still present.

The results of the above studies show that, when using WAAM and additive wire printing, different zones are formed in the workpiece, resulting in different structures and properties. The surface frequently obtains a high hardness. The roughness and surface quality after WAAM are also not too high. Therefore, despite the fact that one of the main goals of additive manufacturing is to reduce production operations, semi-finishing/finishing operations are still required to obtain the desired geometric tolerances and surface properties [8].

The machining of such workpieces requires the consideration of these peculiarities. The milling machining of the workpieces obtained from stainless steels by the WAAM method can be sufficiently highly productive [9]. However, at the same time, significant tool wear was observed when milling the WAAM part. This happens despite the fact that the milling tool and parameters were selected based on the manufacturer’s recommendations for machining such material.

The inhomogeneous microstructure formed along the sample due to complex thermal cycles during WAAM leads to machinability deterioration. A similar pattern was observed when milling with an end mill made of ceramic Al_2_O_3_/Si_3_N_4_ materials (sialon) of the Ti_6_Al_4_V alloy. The workpieces made of this alloy were manufactured using direct metal laser sintering (DMLS). The results of studying the tool after machining such workpieces revealed the presence of the main types of wear [10]. Adhesive wear and diffusion wear are the dominant wear mechanisms, and the chemical resistance of Al_2_O_3_/Si_3_N_4_ ceramics (sialon) is relatively weak in the atmosphere.

In this case, there are several ways to reduce wear and increase tool durability. The first is to apply rational cutting conditions by changing the cutting speed and feed [11,12]. Cryogenically cooling the cutting tool is also an option.

The second is the selection of printing modes, forming the desired surface properties. A methodology for aggregating microhardness data in individual assemblies was developed and tested to overcome the inhomogeneity of the printed workpiece in terms of hardness. A compilation of various AM technologies can also be used. Combining the surfacing processes using an electron-beam (DED), laser LED, and laser fusion in a powder layer (PBF) yields a more homogeneous sample structure in terms of properties [13,14].

Machining the samples, produced by applying two additive manufacturing technologies, demonstrated an increase in cutting forces compared to those of conventional forged workpieces [15]. The samples obtained by laser spraying (LENS) and wire-arc surfacing were equally poorly machined. The results of the work showed a general increase in cutting forces and a decrease in the machinability of both materials obtained via the use of additive technologies. Similar processes taking place during milling were also observed in case of the aluminum alloys obtained via the use of additive technologies. The authors of [16] noted that castings and workpieces obtained via the use of additive technologies provided completely different cutting forces under the same machining conditions.

Climb milling is recommended to machine the parts made of 316 L steel, which is obtained via laser additive-manufacturing (LAM) methods [17]. It provides a better surface roughness quality compared to that obtained via conventional milling. Tool wear can be decreased, and machining productivity can be increased due to the use of ultrasonic vibration milling during climb milling [18]. Vibration application significantly improves the finished surface quality for both titanium alloys and stainless steels [19,20].

Hybrid processes, which combine additive and subtractive (machining by CNC machines) processes (A/SM CNC), i.e., the AT object formation and its subsequent mechanical machining, are performed on the same equipment without idling and are vitally important. They are used to manufacture solid parts from metal powders and/or, when adding some complex elements, to apply special deposited coatings for manufacturing multi-metal parts and for the repair/restoration of worn or damaged expensive parts [21].

The authors of [22] also note the importance of determining the optimal machining conditions of the workpieces obtained via additive wire printing methods. Conventional machining conditions supposedly do not provide optimal results. In general, the authors of [22,23,24,25] also note that the position at which the workpiece is placed during 3D printing helps form different properties. Vertically manufactured workpieces are cooled more slowly than horizontally arranged ones. As a result, depending on the workpiece position when printing, its properties will be different. This will also influence the machining modes. Therefore, when assigning subtractive machining modes, it is important to know the peculiarities of manufacturing the workpiece. This will directly influence the quality of machining and tool wear. An analysis of the literature shows that there are works devoted to the properties of differently oriented printed samples. However, there are practically no works demonstrating how much these changes in the properties of differently oriented printed samples influence the modes of subtractive manufacturing.

There are very few studies devoted to the subtractive processing of workpieces obtained via electron-beam printing. Therefore, the topic of selecting optimal machining conditions for workpieces obtained via wire surfacing methods is extremely relevant. Among the surfacing technologies, electron-beam surfacing ensures the best quality of the material and the least porosity. However, the high costs and complexity associated with the equipment used in electron-beam surfacing limit its usage.

The purpose of this work is to determine the optimal modes for the subtractive machining (milling) of workpieces obtained via the electron-beam surfacing of stainless steel wire.

In our work, we determined the printing modes of two types of samples using wire by means of an electron-beam printer. The samples were printed horizontally and vertically. The study of the structure, properties, and milling modes of such samples is of both practical and scientific interest. Another important aspect of the present study is that it considers the change in the magnitude of the components of the cutting force when cutting with one tooth. This paper reveals and explains the reasons for the strange changes in the milling forces of *P_h_* and *P_v_*, which have been noted by other authors when milling other materials, but no analyses or justifications of these changes have been provided in the existing literature.

After perusing numerous publications in the existing literature, we can confirm that the designation of the components of the cutting force *F_z_*, *F_y_*, and *F_x_* was most often used. The direction of these forces corresponds to the direction of forces during the turning process yielded by the Kistler dynamometer software to the monitor. In fact, it is necessary to use the designation of the force in the direction of the *P_h_* supply and the lateral force *P_v_*. These forces reflect the magnitude of the forces regardless of the feed direction and the dynamometer position on the milling machine table. Forces *F_y_* and *F_x_*, which are reflected on the monitor during milling, depend on both the position of the dynamometer on the milling machine table and the feed direction during milling.

## 2. Materials and Methods

The experimental work consisted of three stages. In the first stage, samples were obtained by using an electron-beam unit. At this stage, the printing modes to obtain the two types of the samples (vertical and horizontal) were developed. Vertical samples in the form of a column were 70 × 15 × 14 mm (height × width × length), and horizontal ones were 14 × 70 × 15 mm. In the second stage, the microstructure and hardness were studied for these samples. Finally, in the third stage, we analyzed the influence of milling modes on the cutting forces arising during machining.

To produce the samples, 40 × 13 stainless steel (an analog of the American AISI 420 or European X41Cr13 steel) was used. The chemical composition of this steel is provided in Table 1.

### 2.1. Production of Samples Using an Electron-Beam Unit

**Figure 1:**
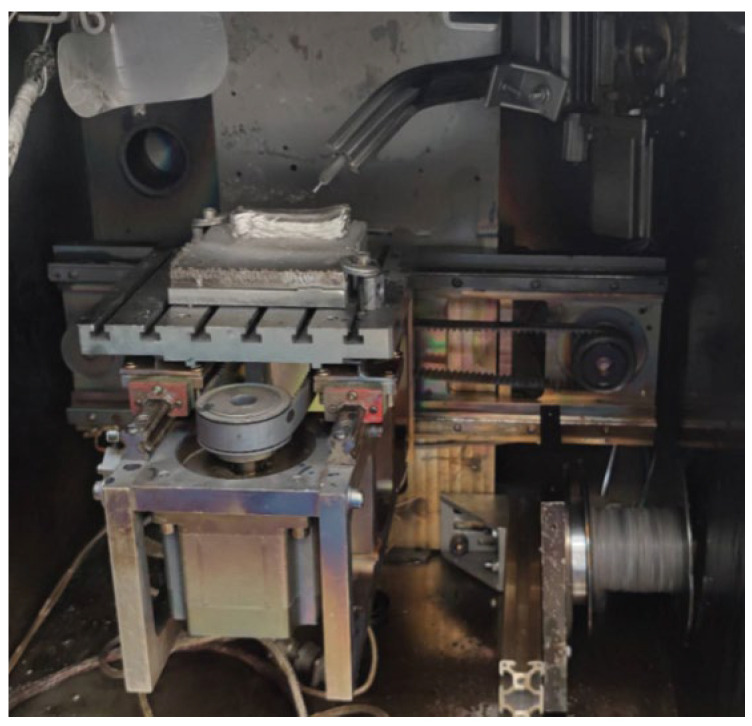
General view of the working chamber of the electron-beam unit for wire surfacing.

The samples were printed using an electron-beam unit (EBU) for wire surfacing. The unit was designed and manufactured at Tomsk Polytechnic University (Figure 1).

The accelerating voltage of the EBU was 40 kV and remained unchanged. The current variation range was within 0–200 mA. The initial material intended for obtaining the workpiece by the EBU method was 420-grade steel wire with a diameter of 1.2 mm. The chemical composition of the wire is presented in Table 1.

**Figure 2:**
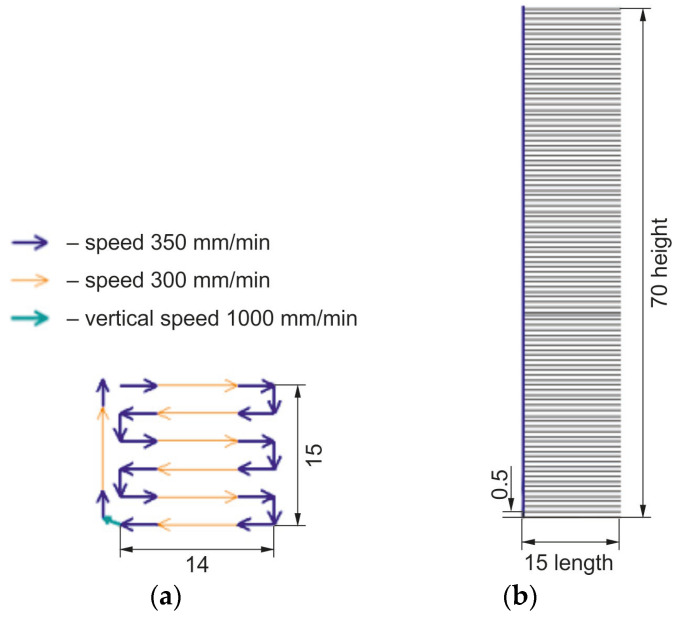
Scheme for printing a vertical sample: (**a**)—scheme for printing a single horizontal layer; (**b**)—scheme for printing vertically.

**Figure 3:**
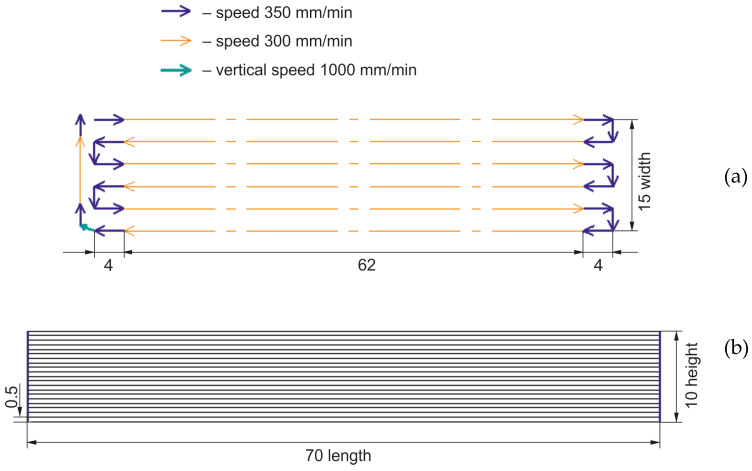
Scheme for printing a horizontal sample: (**a**)—scheme for printing a single horizontal layer; (**b**)—scheme for printing vertically.

Two batches of the samples were produced to conduct mechanical tests: vertical and horizontal. The dimensions of the print trajectory for the horizontal samples were 70 × 15 × 14 mm (Figure 2). The sizes of the print trajectory for the vertical samples were 14 × 15 × 70 mm (Figure 3).

The following modes were used when printing the samples:

- Circle beam sweep: 3–5 mm;
- Accelerating voltage: 40 kV;
- Wire feed angle: 45°;
- Beam current for a vertical sample: 21 mA;
- Beam current for a horizontal sample: 30 mA;
- Wire feed rate for a vertical sample: 700 mm/min;
- Wire feed rate for a horizontal sample: 1050 mm/min.

When printing, the same material (steel 40 × 13) used for the wire was used as the substrate material. Printing was carried out in a vacuum at a pressure of 5 × 10^−3^ Pa.

### 2.2. Investigation of the Microstructure and Hardness of the Obtained Samples

**Figure 4:**
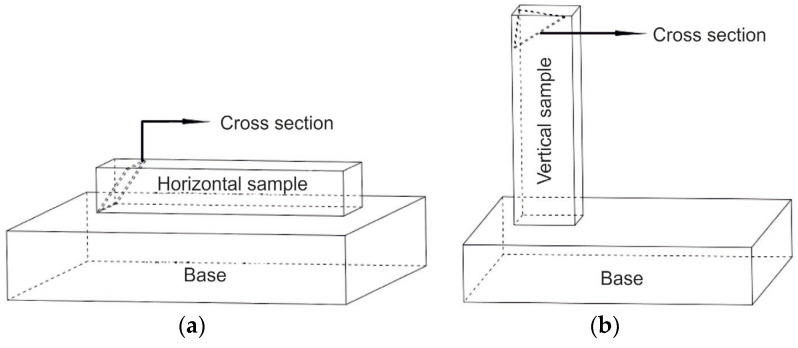
Scheme for studying the microstructure of samples: (**a**)—horizontal samples; (**b**)—vertical samples.

To study the microstructure of the samples, oblique sections of the samples were made, as shown in Figure 4. Using the oblique cross-section allows for the immediate detection of possible defects formed in the workpiece’s various deposited coatings (for both the vertical and horizontal samples).

The microstructure was etched using an etcher consisting of a mixture of concentrated nitric HNO_3_ (67 wt.%) and hydrochloric HCl (33 wt.%) acids taken in a ratio of 1:3 by volume. Microstructural studies were carried out using an MMP-1 metallographic microscope manufactured by BIOMED. The microstructure photo was obtained using the DCM-510 SCOPE video eyepiece. The microhardness was measured using an automatic complex based on the “EMCO-TEST DuraScan-10” microhardness meter. Measurements were taken for the same samples that underwent metallographic study. Measurements were carried out using a Vickers indent under a load of 1 kgf at a shutter speed of 10 s.

### 2.3. Studying the Cutting Forces during Milling

The printed samples were milled on a CNC machine (CONCEPTMill 155; purchased from the EMCO Company). The cutting forces were determined using a Kistler 9257 V dynamometer (Switzerland) (Figure 4). The data were analyzed using DynoWare software. Several types of mills were selected as a tool. The dynamometer sensitivity was 7.5 N; the measurement error was ±0.005%. When measuring the cutting forces in various experiments (in case of the reinstallation of the workpiece or mill) in the presence of certain parameters, the spread was no more than 15%. When machining by cutting, this indicates fairly good repeatability, taking into account the complexity of the cutting process itself and the heterogeneity of the sample properties in different zones. Errors can occur when adjusting to the required depth and width of milling. During our experiments, even a small amount of wear on the mill was identified.

Two-lip end mills made of high-speed P6M5 steel manufactured by the ZCC-CT company (China) were used. This steel is a high-carbon steel containing 0.85% of C, 5.5–6.5% of W, 4.8–5.3% of Mo, ≈80% of Fe, 0.85–0.9% of C, 3.8–4.4% of Cr, and 1.7–2.15% of Va (Co < 0.5%, Mn < 0.5, Si < 0.5%, Ni < 0.4%, S < 0.025%, *p* < 0.025%). These two-tooth phrases had a diameter of 8 mm. The helical groove angle (helix angle) was ω = 35°, the radial rake angle was 7°, the primary clearance angle was 5 º, the cut length was 25 mm, and the overall length was 45 mm. The two-tooth mills were chosen because when the milling depth t is less than half the diameter of the mill (t < D/2), only one tooth of the mill is capable of cutting. It is possible to study the change in the magnitude of the cutting forces over time when milling with only one tooth. When selecting the cutting modes, we used both our own exploratory studies and literature data [26,27].

**Figure 5:**
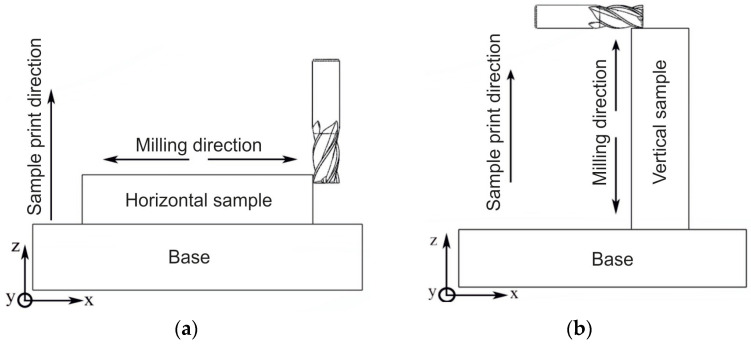
Milling scheme for: (**a**)—horizontal samples; (**b**)—vertical samples.

**Figure 6:**
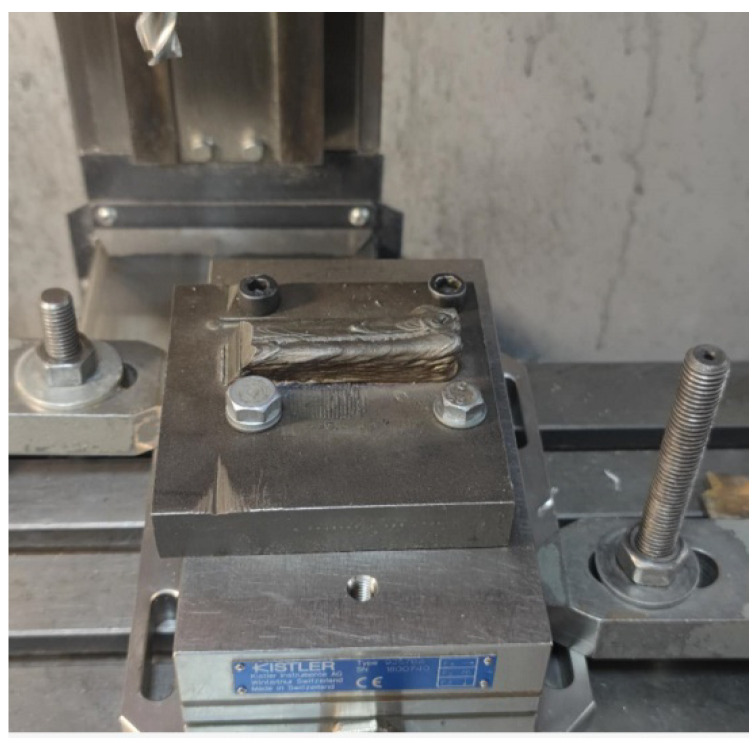
General view of the horizontal sample fixed on the machine.

For both sample types, Hard-alloyed end mills with diameters of 8 and 12 mm, produced by the GESAC company (China), were also used. The hard alloy mainly consisted of tungsten carbides (~92%) and a cobalt bond (~8%). Their parameters are presented in Table 2. Coated mills were used. The mill coating had a high hardness, low friction coefficient, and high resistance to oxidation (Table 3). The mills with a diameter of 8 mm had 4 teeth, and the mills with a diameter of 12 mm had 6 teeth. The helical groove angle was ω = 35°, the rake angle was 7°, and the relief angle was 5°. The choice of the mills’ coating is determined by the machining conditions. Dry milling was used in the experiments. Various milling schemes were used for the vertical and horizontal samples. These schemes are shown in Figure 5. The samples were fixed on the machine along with the substrate, as shown in Figure 6.

We milled the tested samples in the same modes 3 times a day at different times. We did not consider the difference in strengths because each tooth has its own strength due to radial beats. Upon changing, the largest value for each component was selected. When the mill rotates, many peaks enter the time interval. Even within 5 s of the measurement, about 14–15 peaks are obtained for each tooth. Based on this, at least 15 measurements were made per approach while milling. Upon analyzing the resulting data, we obtained an average value and used it to plot graphs.

**Figure 7:**
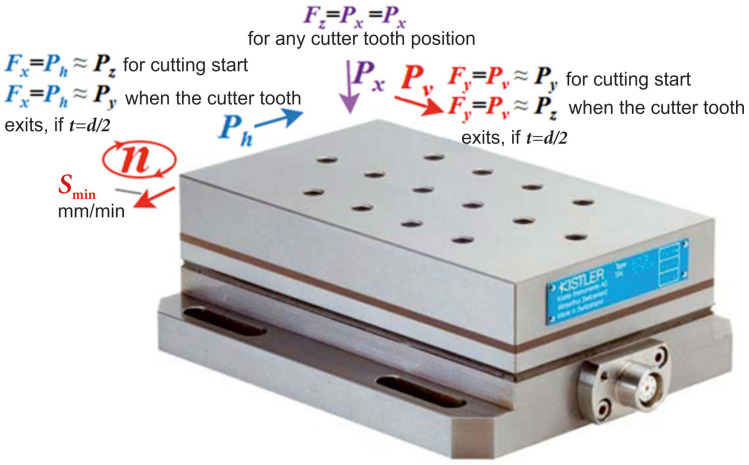
Directions of forces *Fx*, *Fy,* and *Fz*, measured using the Kistler dynamometer.

**Figure 8:**
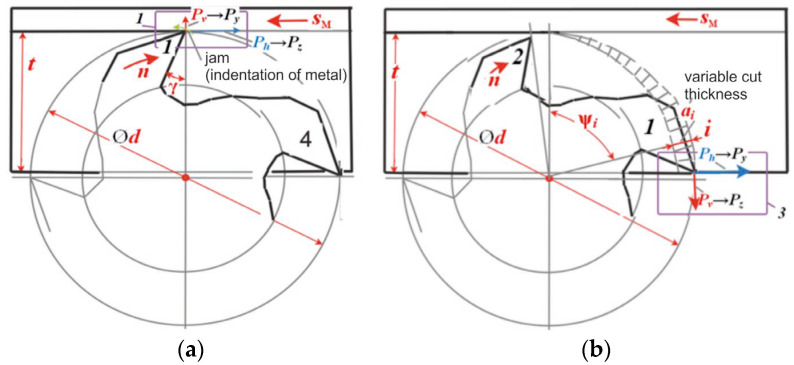
Scheme of the direction of the cutting force components during asymmetric conventional milling: (**a**)—when tooth No. 1 is embedded; (**b**)—when tooth No. 1 is withdrawn from the contact with the workpiece.

Kistler dynamometer software allowed us to establish the fact that the vertical force on the monitor is marked by the *Fz* symbol, i.e., as in the turning machining. When milling, the vertical direction of the force *Fz* action corresponds to the axial force *P_x_* (Figure 7 and Figure 8). Figure 7 shows that the directions of the measured forces *Fx*, *Fy*, and *Fz* displayed on the monitor by the Kistler dynamometer correspond to the milling forces *P_h_*, *P_v_*, and *P_x_*. In addition, the forces *Fx*, *Fy*, and *Fz* approximately correspond to the tangential *P_z_* and radial *P_y_* forces when cutting down and when the tooth ceases to make contact, if *t* = *d*_m/_2 (Figure 1 and Figure 2). The scheme of the direct action of these forces on a four-tooth end mill with a diameter of *d* = 8 mm at a milling depth of *t* = *d*/2 − 0.2 mm = 3.8 mm is shown in Figure 8. The figure shows 2 moments: when tooth No. 1 was embedded after tooth No. 4 had already been withdrawn from making contact (shown in Figure 8a) and when tooth No. 1 was withdrawn from making contact with the workpiece before tooth No. 2 had come into contact with the workpiece (shown in Figure 8b).

When the sample is milled using the feed direction across the dynamometer or along the workpiece when it is fixed with its long part across the dynamometer, the *F_x_* force direction on the dynamometer monitor corresponds to the *P_h_* force direction (the force directed against the feed direction of the platen *S* (mm/min) during conventional milling). At a shallow milling depth (*t* << D), the *P_h_* force approximately corresponds to the *P_z_* force acting on the mill tooth.

If *t* = D/2, when the tooth is embedded, the *P_h_* force approximately corresponds to the *P_z_* force, i.e., to the tangential component of the cutting force. Additionally, when the tooth is withdrawn from making contact with the workpiece, it already corresponds to the *P_y_* force acting on the mill tooth in the radial direction, i.e., to the axis of the mill.

The direction of the force *F_y_* displayed on the dynamometer monitor corresponds to the direction of the force *P_v_* (the force directed perpendicular to the feed direction of the platen S) when milling the sample using the feed direction across the dynamometer, i.e., along the workpiece during its fixing with its long part across the dynamometer. At a shallow milling depth (t << D), the *P_v_* force approximately corresponds to the *P_y_* force acting radially on the mill tooth. If (t ≈ D/2), when the tooth is embedded, the *P_h_* force approximately corresponds to the *P_y_* force, and when the tooth is withdrawn from the contact with the workpiece, it already corresponds to the *P_z_* force acting on the mill tooth.

When calculating the mill strength, forces equal in absolute value but opposite in sign are applied to its tooth, i.e., +*P_z_* from the tooth to the workpiece = –*P_z_* to the tooth on the workpiece part. When we say that *P_v_* corresponds to the *P_y_* force acting radially on the mill tooth, we consider the force magnitude by the absolute value *P_y_* = |*P_y_*_dyn_| = |*P_y_*_tooth_| because we cannot measure the *P_y_* force acting on the mill tooth, only that acting on the dynamometer.

## 3. Study of the Influence of the Modes for Obtaining Samples via Electron-Beam Surfacing on Their Structure and Properties

In the first stage of the experiment, two types of samples were produced: samples printed vertically and those printed horizontally. A detailed description of the technology of manufacturing samples is provided in Section 2 (“Materials and Methods”). Since the technology involved in printing the samples via electron-beam surfacing with wire is quite new, there are practically no standard modes of sample production. The printing modes will mainly depend on the printed material and the geometric dimensions of the samples. Therefore, in this case, preliminary experimental work was required to determine the printing modes for the production of the samples we used in our equipment. When choosing the initial values of the modes, we were guided by our experience of similar work regarding the printing of samples similar in size to that of the workpiece and the wire diameter. We introduced a small spread of the beam current value to determine the optimal machining modes. When conducting the experiments, six different values of the beam current were used. The value at which the highest quality sample was obtained was then used to obtain the next three samples. Then, the experiments were conducted using these samples to determine the cutting forces.

### 3.1. Determination of Surfacing Modes and Sample Production

Several experimental samples were printed in this study.

**Figure 9:**
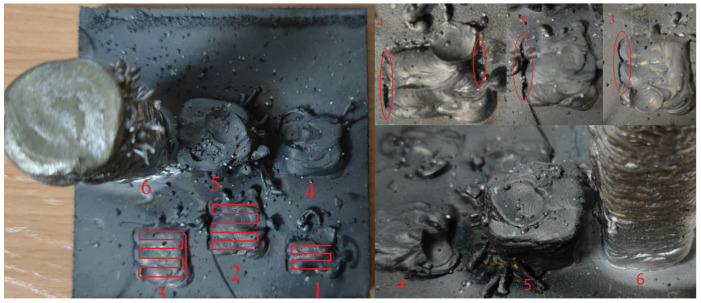
Images from the experiment focusing on obtaining a vertical sample. Beam current values for the samples were as follows: 1—33, 2—30, 3—27, 4—25, 5—23, 6—21 mA.

Different beam current values were used (33, 30, 27, 25, 23, 21 mA) for the vertical samples (Figure 9).

The first printed layer had the highest cooling rate. When a beam current was more than 25 mA, it was not possible to successfully print even the first layer. This beam current was too large, thus melting the wire and the substrate material. As a result, a cavity appeared on the border of the printed sample. Printing the subsequent layer was not possible. As the beam current decreases, the printing path length increases. When the beam current is 25 mA, 4 deposited coatings were successfully printed; 13 deposited coatings were printed at 23 mA. When the number of printed deposited coatings increases, the cooling rate decreases, and the total temperature of the sample increases. If there is no time to remove heat from the sample, then the wire is in a liquid metal state, enabling it to move around the melting zone. The optimal beam current for the vertical sample had a value of 21 mA. At the same time, the wire was fed at a speed of 1050 mm/min. For the horizontal samples, the area of contact with the substrate is much larger. As a result, the heat accumulation in the printed sample is slower. This fact allows for the printing of a sample under a higher beam current. For the horizontal samples, the optimal beam current value was 30 mA at a wire feed rate of 700 mm/min, and faster solidification, a higher layer printing rate, and a lower wire feed rate all lead to a higher printing accuracy.

### 3.2. Study of the Microstructure and Microhardness of the Printed Samples

In conventional production methods, martensitic stainless steels are usually completely martensitic. If samples are manufactured using additive technologies, other phases, such as austenite and delta ferrite phases, can be detected in them.

**Figure 10:**
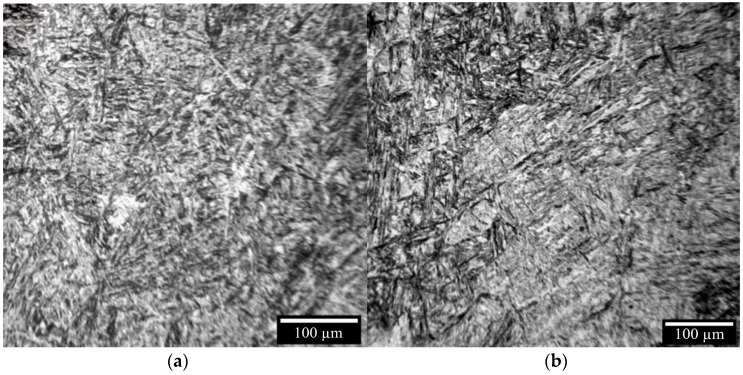
Microstructure of: (**a**)—horizontal samples; (**b**)—vertical samples.

Figure 10 shows that the printed samples have a completely dense structure without the crack formation at the interlayer boundaries. There is no molten bath boundary. This indicates the material has melted well during the printing process.

The microstructure of the printed samples is similar to the microstructure of the 40 × 13 steel after quenching and low tempering [28,29]. Figure 10a,b shows the martensite with a needle-like structure. This behavior is explained by the high cooling rate during solidification in the case of electron-beam additive manufacturing, which facilitates the phase transformation of austenite into martensite. These martensitic needles with a random orientation are much smaller than the martensitic needles formed during the casting and quenching of the 40 × 13 stainless steel [30,31]. During the additive printing of the sample, the heat released by the electron-beam to form the subsequent layer significantly influences the final microstructure of the previously deposited coatings. When applying the layer, the lower layer near the upper layer is heated at a temperature that is above the austenitization temperature. As a result, the previously formed martensite turns back into austenite. After re-cooling, residual austenite and martensite are formed. In the deposited coatings that have not reached the austenitization temperature, the temperature may be high enough to start the process of martensite tempering. Part of the residual austenite in these deposited coatings will turn into martensite. It is worth noting that the martensitic needles in the vertical samples (Figure 10a) are larger than those that are in the horizontal samples (Figure 10b). This is also due to their different cooling rates.

**Figure 11:**
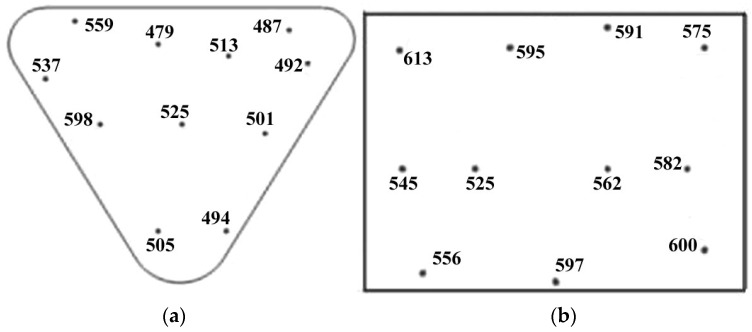
Microhardness of: (**a**)—horizontal samples; (**b**)—vertical samples.

Our analysis of the samples’ hardness (Figure 11) showed that, for the vertical samples, the average cross-sectional hardness was 517 HV (Min—479 HV; Max—598 HV). For the horizontal samples, the average cross-section hardness was 576 HV (Min—525 HV; Max—600 HV). The horizontal samples had higher cooling rates during the manufacturing process; therefore, they formed a more uniform distribution of needle-like martensite. In the case of the horizontal samples, the cooling rate was lower, and a greater amount of residual austenite was formed. As a result, the hardness of these samples turned out to be lower. The hardness values of the obtained samples correspond to the hardness of the 40 × 13 steel after quenching and low tempering (HRC50-55) [28,29].

## 4. Study of Cutting Forces when Machining the Samples

In the third stage of our experiment, we studied the cutting forces that occur when milling the printed horizontal and vertical samples. When machining the printed samples, great attention was paid to the component forces *P_h_* and *P_v_*. This is because the *P_x_* force is relatively small and is directed along the mill axis. In this direction, the rigidity of the end mill is very high. In the radial direction, the end mill rigidity is not great. Therefore, a large value of the *P_y_* force acting radially to the mill axis leads to vibration.

To study the cutting force components when machining the manufactured samples, different feeds were used in each series of the experiments (Table 4).

**Figure 12:**
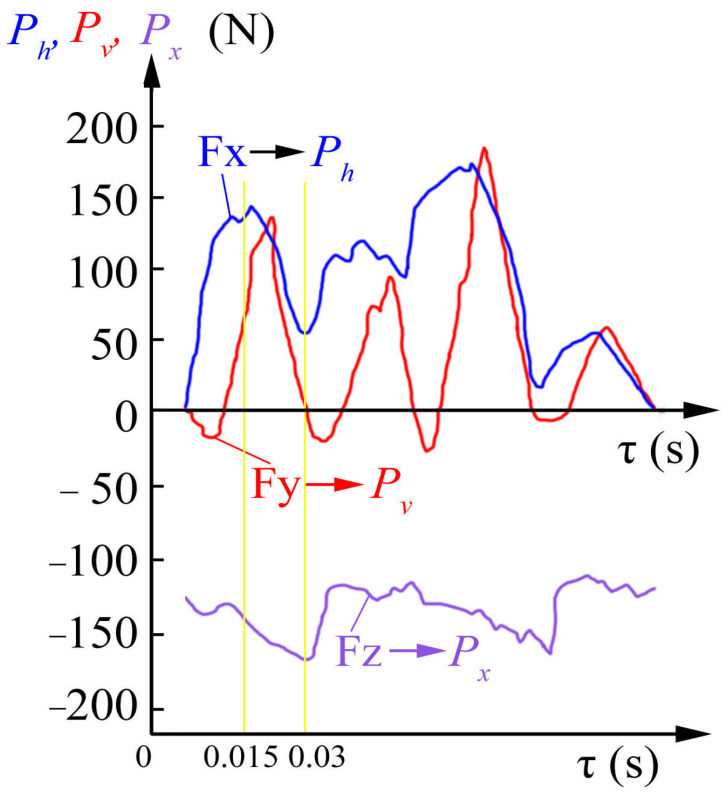
Graphs of changes in forces when turning a sharp mill per revolution. Conventional milling with a four-tooth unworn mill with Ø*d* = 8 mm, *t* = 3.8 s, *b* = 2 mm, *n* = 500 rev/min, *s*_m_ = 28 mm/min. Stainless steel with a diameter of 40 × 13 (Vertical sample) is the VK8 hard alloy.

Firstly, a graph of the change in the cutting forces when milling with a four-tooth mill was built. Figure 12 shows the change in the components of the cutting forces depending on time τ (s) when milling with the four-tooth mill with a diameter of 8 mm when *t* = 3.8 mm. A mill diameter of slightly less than 4 mm was taken to ensure that the subsequent tooth did start cutting prematurely. The graph shows the forces that arise during one full revolution of the mill. Four distinct peaks are visible on the curves of the principle components of the cutting forces *P_h_* and *P_v_*. These peaks correspond to the work of each of the teeth of the mill. The force increases when the tooth is embedded in the workpiece, and the force decreases when the tooth leaves the cutting zone.

The graphs (Figure 12) of the change in the magnitude of the *P_h_*, *P_v_*, and *P_x_* components of the cutting force demonstrate that their magnitude varies non-synchronously. This is explained by the turning of the *P_z_* and *P_y_* forces during the mill rotation. The magnitude of the *P_h_* and *P_v_* forces is a consequence of changes in the magnitude and direction of the *P_z_* and *P_y_* forces. The tangential *P_z_* force of an unworn mill has the greatest magnitude. This happens due to the fact that it participates in cutting a metal layer with a variable cut thickness *a_i_ = s_z_∙sinψ_i_*, where *s_z_* is the feed per tooth (mm/tooth); ψ*_i_* is the instantaneous value of the central angle of the tooth position (contact angle) (°—degrees) (Figure 8b). When the mill teeth are worn along the back surface, the radial *P_y_* force increases significantly and becomes two to three times greater than the *P_z_* force, depending on the wear degree of the mill tooth.

The graphs in Figure 12 were recorded over a short period of time ( 5–7 s). Recording the changes in the forces over a longer period of time (more than 30 s) to reveal a trend regarding a change in the magnitude of the components of the cutting force to establish a stable temperature regime does not allow one to isolate the changes in the forces that occur during cutting with each tooth. Therefore, the largest magnitude of each component was recorded according to peaks on their graphs.

### 4.1. Influence of the Cutting Speed and Feed on the Force Components during Milling

**Figure 13:**
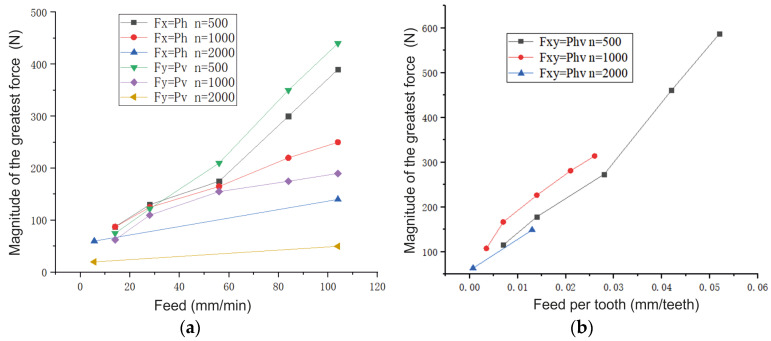
Influence of feeding (**a**) and feeding per tooth (**b**) on the magnitude of the greatest force during conventional milling; B = 2 mm, t = 4 mm, d = 8 mm. A vertical sample is mill No. 1.

When *n* = 500 rpm and *s*_m_ ≥ 56 m/min, the cutting force components increase intensively. In addition, the low end speed (*n* = 500 rpm) of the spindle and the large feed per tooth (*s_z_* = 0.052 mm/tooth) lead to intense wear and chipping along the tops of the carbide mills (Figure 13). When *n* = 2000 rpm and the feed increases from 5.6 to 104 mm/min, the resulting cutting force F_xy_ increases up to 2.35 times.

**Figure 14:**
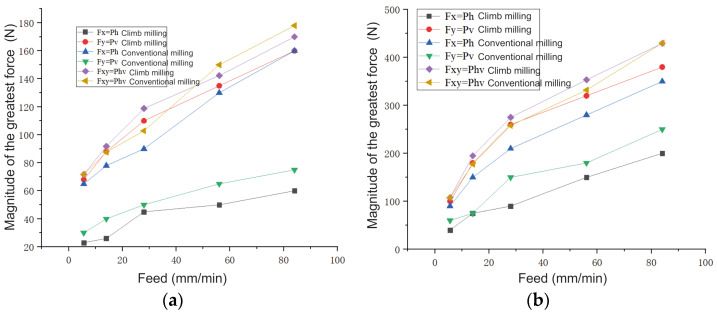
Influence of the feed *s*_m_ per minute on the largest value of the component forces. A horizontal sample is mill No. 2; *d* = 8 mm, *t* = 5.5 mm; *B* = 2 mm. (**a**)—*n* = 2000 rpm, (**b**)—*n* = 500 rpm.

During high-speed milling at a higher rotational speed (*n* = 2000 rpm), the feed does not significantly affect the lateral cutting *P_v_* force (Figure 14a), but the *P_h_* force directed against the feed direction increases more intensively.

An increase in spindle speed from 500 to 2000 rpm when a value of the minute feed *s*_m_ is constant leads to a significant decrease in the magnitude of the greatest cutting force. The cutting force is reduced not only due to reducing the feed per tooth but also due to a reduction in the contact time regarding each tooth and the workpiece and chips. The short contact time (when *n* = 2000 rpm and z = 4 pcs, τ = 0.0075 s) and the high rate of chip flow lead to a large amount of heat, which is released in the cutting zone, being removed, along with the chips. Therefore, the workpiece and the tool do not have enough time to heat up.

### 4.2. Conventional and Climbing Milling of the Studied Samples

Figure 14 demonstrates that the cutting *P_h_* force during conventional milling is significantly greater than that during climb milling. However, the *P_v_* force during the conventional milling of the cutting is significantly less than that in the case of climb milling. Therefore, the value of the resulting cutting *P_hv_* force during conventional and climb milling is almost the same.

When the spindle speed is high, the difference between the magnitudes of the components of the cutting forces decreases (Figure 14):

(1) When feed per minute is large, e.g., *s*_m_ = 84 mm/min, the difference of the component forces during climb and conventional milling at *n* = 500 rpm is ∆*P_h_* = 200 − 350 = −150 N and ∆*P_v_* = 380 − 240 = 140 N; at *n* = 2000 rpm it is ∆*P_h_* = 55 − 160 = −90 N and ∆*P_v_* = 160 − 75 = 85;
(2) When feed per minute is small, e.g., *s*_m_ = 14 mm/min, the difference of the component forces during climb and conventional milling at *n* = 500 rpm is ∆*P_h_* = 30 − 80 = –50 N and ∆*P_v_* = 90 − 52 = 38 N; at *n* = 2000 rpm it is ∆*P_h_* = 22 − 62 = –40 H and ∆*P_v_* = 68 − 25 = 43 N.

Climb milling is appropriate for finishing milling when the crust on the sample has been previously removed. However, in the case of wire additive technologies, the initial surface unevenness and the allowance are greater than those obtained via powder additive technologies. During conventional milling, the load per tooth gradually increases. Additionally, although the cutting resistance in the feed direction (*P_h_* force) increases in comparison with climb milling (Figure 14), its direction against the direction of the platen feed chooses the side gaps in the screw pair “platen feed screw—platen nut”, which prevents jerks when the next tooth of the mill penetrates and reduces vibration.

The bending moment acting on the mill on the part of the *P_h_* force during conventional milling increases compared to climb milling; however, the *P_v_* force decreases by almost the same amount (Figure 14). This increases the accuracy of machining.

**Figure 15:**
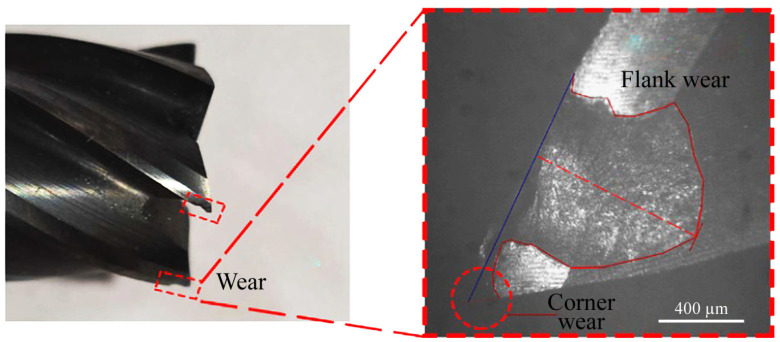
Spalling along the main cutting edge of the mill tooth when machining the sample crust.

As noted earlier, the samples have a high hardness. Therefore, during climb milling, the tooth hits it, which leads to the rapid destruction of the cutting edge, especially on the corners. When machining uneven surfaces with a milling width of *B* > 2 mm, the zone of the greatest spalling is not on the corners of the carbide mill tooth but on the main cutting edge (Figure 15). An uneven surface leads to an uneven cutting process; therefore, the machines with low rigidity are exposed to the vibration, which is so large that the screws and nuts on the spindle head are spontaneously unscrewed.

### 4.3. Influence of Milling Width on Cutting Forces

**Figure 16:**
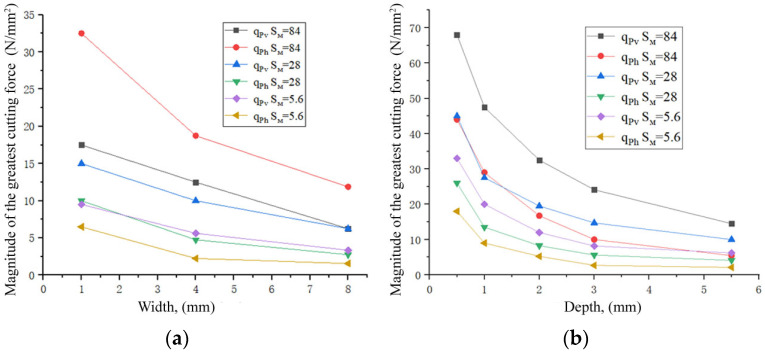
Influence of the width ((**a**), *t* = 2 mm) and depth ((**b**), *B* = 2 mm) of milling on the magnitude of the largest specific components of the *P_v_* and *P_h_* forces at different values of the feed per minute *s*_m_. A horizontal sample is mill No. 2; *d* = 8 mm, *n* = 2000 rpm.

An increase in the width *B* and the milling depth *t* leads to a gradual decrease in the specific cutting force (Figure 16a). It is recommended to use milling with a large width and the same volume as the cut layer. When milling using a large width, the temperature of the cutting edge of the mill tooth is lower and more evenly distributed along the cutting edge due to the improved dissipation of heat into the mill body. In addition, the bending moment of the mill is also reduced due to the distance between the zone of applying *P_h_* and *P_v_* forces to the end of the collet chuck, in which the end mill with a fixed diameter of less than 16 mm decreases.

When the milling depth t increases, the specific cutting forces first decrease sharply, then the intensity of their reduction decreases (Figure 16b). Increasing the milling depth *t* from 3 to 5.5 mm ensures the teeth of the mill are always in contact with the workpiece (working), and the unevenness of the cutting process and the impact load are reduced. To achieve a higher material removal rate and a low specific cutting force, milling using a width of *B* = 6 mm (*t* = 0.5 mm in case of a mill diameter exceeding 12 mm) is recommended.

### 4.4. Influence of the Mill Teeth Number

**Figure 17:**
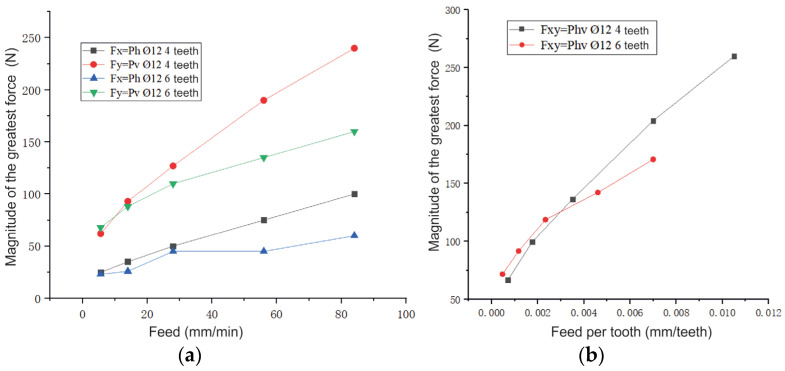
Influence of the feed per minute *s*_m_ (mm/min) (**a**) and the feed per tooth *s*_z_ (mm/tooth) (**b**) on the magnitude of the resultant force *P_hv_* (H) during climb milling. *d* = 12 mm, *n* = 2000 rpm, *t* = 5.5 mm, *B* = 2 mm, for a horizontal sample.

When feeding on a S_Z_ tooth_,_ *s*_z_ ≤ 0.003 mm/tooth, the resulting cutting force *P_hv_* of a six-tooth mill is slightly greater than that of a four-tooth mill (Figure 17b), but when *s*_z_ > 0.003 mm/tooth, it is less than that of a four-tooth mill. Additionally, when the feed per tooth is increased, this difference increases (Figure 17b).

If the feed per minute values are the same, the cutting forces *P_h_* and *P_v_* of the six-tooth mill are less than those of the four-teeth mill. Specifically, a big difference can be observed in the *P_v_* force, which increases along with the increase in the feed per minute (Figure 17a). The cutting force reduces due to a reduction in the feed per tooth.

When the feed per minute is constant and the number of teeth increases, the temperature of each mill tooth decreases not only due to a reduction in the feed per tooth but also due to more efficient cooling. This happens because the heat released during cutting moves into the mill tooth, which idly cools until the workpiece is next cut into.

When the number of mill teeth increases, the roughness of the treated surface decreases, and the unevenness of the cutting process is reduced. The allowance for the parts made using powder additive technologies is known to be lower than that in traditional production. The rough machining of the workpieces requires the diameter of the mill to be two times greater than the thickness of the machining allowance (when milling, this will be the milling width *B*).

To machine the parts after AT, it is recommended to use end mills with a large number of teeth (*z* > 4 pcs.). In addition, it is desirable to use high-speed milling (HSM) because this not only increases the machining performance but also significantly reduces all the components of the cutting force. Reducing the feed per tooth leads to a decrease in the volume of the chips to be removed, which should be less than the volume of the end mill groove, determined by its depth *H*. Therefore, reducing the feed per tooth enables the use of the end mill with a large number of teeth.

In general, these recommendations coincide with the recommendations reported elsewhere [7,8,9,10,14,15,16,17,18,19] regarding the machining of workpieces obtained from metal materials via the use of additive technologies. The authors of the above-listed works devoted to printing the workpieces made from heat-resistant alloys and steels also recommend using a carbide tool (mills, cutters) for machining. When machining the printed workpieces, described both in our manuscript and in the works of other authors, using the maximum possible cutting speeds is recommended. However, at the same time there will be a cutting speed limitation, which is caused by the maximum speed of the mill rotation. The value of this maximum speed is up to 350–500 m/min, and it depends on the hardness of the processed material and the wear-resistant coating of the milling surface. Using replaceable multifaceted plates that have a mechanical attachment to the mills facilitates an increase in both the tool diameter and maximum speed. However, at the same time, vibration increases, and the milling unevenness and the processed surface quality deteriorate.

### 4.5. Influence of the Mill Material

**Figure 18:**
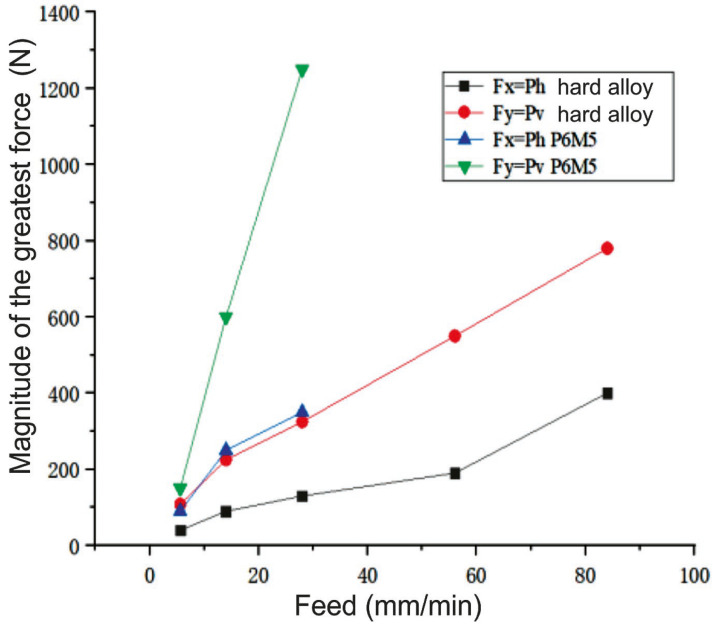
Influence of the feed per minute *s*_m_ (mm/min) and the mill material on the magnitude of the greatest *P_h_* and *P_v_* forces during climb milling. A horizontal sample; *d* = 8 mm, *n* = 500 rpm, *B* = 2 mm, *t* = 3.8 mm.

**Figure 19:**
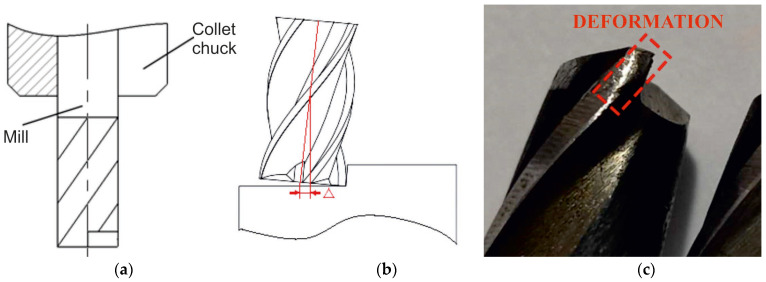
(**a**) Scheme of the resulting *P_hv_* force action. (**b**) The deviation of the mill from the vertical position. (**c**) The plastic deformation of the cutting edge of the high-speed mill made from P6M5 (*d* = 8 mm).

High-speed steel mills are not suitable for machining EBM 420 SS parts due to their intense wear associated with the high hardness not only of the crust but also of the metal in the workpiece depth, as well as the fact that the hardness of high-speed steel (for example, P6M5, P6M5K5, etc.) is insufficient. As the feed increases, the magnitude of the greatest force at the mill made from P6M5 increases sharply (Figure 18). The friction force between the back surface and the machined material will increase significantly [20]. The friction coefficient of the uncoated mill from P6M5 is greater than that of the carbide mills. The absence of CCF leads to an increase in the cutting temperature. Wear and the plastic deformation of the cutting edges (Figure 19c) are the result of high cutting temperatures.

Table 5 shows that, for a hard alloy mill, as the feed increases, the ratio between the decrease time and the increase time reduces. When the feed per minute is *s*_m_ = 84 m/min, the time ratio is 1.18, which means that the mill bends strongly and deviates from the vertical position (Figure 19a,b). A low spindle speed, a large feed per tooth, and the use of climb milling lead to an increase in *P_h_* and *P_v_* forces, which causes the inclination of the mill from the vertical position (Figure 19a,b). This can also cause the mill to break, a deterioration of the treated surface quality, and a decrease in accuracy.

From our experimental work, we determined the forces acting on a tool when machining the samples made from heat-resistant steel obtained via the EBW wire method. Regularities of variations in cutting forces when cutting with one tooth were also obtained. Important results include determining the change in the direction of the lateral component of the cutting force *P_v_*. If there is a gap in the screw pair of the machine, this leads to vibration. Therefore, machining such workpieces requires the careful preparation of equipment. If there is a gap in the screw pair of the transverse feed, then there is no need to reduce it to a minimum. It is also worth noting that when the width increases by more than 3 mm of milling *b*, the change in the direction of the lateral force *P_v_* is terminated. Knowing the *P_h_* feed force, which we were able to achieve through the results of the conducted tests, allows for the calculation of the required strength of workpiece-retaining pressure. This is especially important if the workpiece has low rigidity. These results are crucial for manufacturing enterprises engaged in machining workpieces obtained via the EBW wire method.

## 5. Conclusions

The cutting forces were studied by milling rectangular-shaped samples made by the electron-beam surfacing of martensitic stainless steel. Based on our experiments, the following conclusions were made:

1. Tempered martensite is observed on the selected sections. The average hardness of the samples is similar to the hardness after quenching and tempering (576 HV for horizontally printed blanks and 525 HV for vertically printed blanks).
2. The resulting cutting *P_hv_* force when machining different samples under the same cutting modes did not change significantly. When machining horizontally arranged samples, the components of the milling force are 5% greater than those are when milling vertical samples, which is explained by a higher cooling rate and, as a result, a slightly higher hardness (51HRC versus 50HRC for the samples obtained during vertical synthesis).
3. When the feed per minute increased from 5.6 to 104 mm/min, the resulting cutting *P_hv_* force increases 2.35 times.
4. The value of the resulting cutting *P_hv_* force during climb and conventional milling is almost the same. However, the cutting force in the direction of the *P_h_* feed during conventional milling is significantly greater than that in case of climb milling, and the lateral *P_v_* force during conventional milling is significantly less than that in case of climb milling.
5. An increase in the milling width leads to a gradual decrease in the specific cutting force. As the milling depth increases, the specific cutting force decreases very intensively at first, but then it decreases more slowly.
6. The resulting cutting *P_hv_* force of a six-tooth mill is less than that of a four-tooth mill. Additionally, when the feed per tooth increases, this difference becomes greater.
7. The machining of the workpieces made of 40 × 13 steel formed using additive technology via electron-beam melting (EBM) (EBM 420 SS) requires using purely carbide mills of a diameter of at least 12 mm. Such mills must have the largest possible number of teeth, the highest possible cutting speed (but not exceeding 300 m/min), and a feed per tooth of *s_z_* = 0.007 − 0.012 mm/tooth. The milling width should be no more than the diameter of the mill, and the milling depth at the same time must be no more than 0.2 of the diameters of the mill. It is better to increase the milling width to maintain the same cross-sectional area (*B* × *t*) of machining. The greater the rigidity of the machine and the fixture, the more favorable the machining conditions and the quality of the machined surface.
8. During our research, we used the standard method of changing the cutting forces by means of a dynamometer. However, to determine the forces *P_z_* and *P_y_*, we observed a number of conditions. The milling depth t was slightly less (by 0.2 mm) than half the diameter of the mill *d* (*t* ≈ 0.5∙*d −* 0.2), and a two-toothed (z = 2) or four-toothed (z = 4) mill was used. The milling width *b* is not more than 2 mm. This allowed us to determine the forces *P_z_* and *P_y_* and use them to determine the physical components of the normal N and tangential F forces on the front surface of the mill tooth, taking into account the front rake. If one knows the physical components on the front surface of the tooth, one can plot epures of contact stresses. This is especially important when designing and calculating the mill in terms of the strength of the wedge. In this paper, we applied this technique to determine the forces *P_z_* and *P_y_* acting on the mill tooth when machining heat-resistant steel. In our future work, we will aim to identify the forces *P_z_* and *P_y_* when machining other heat-resistant materials (titanium alloys) obtained via the EBW wire method.

## Figures and Tables

**Table 1:** Chemical composition of the 40 × 13 stainless steel.

C	Si	Mn	Ni	S	P	Cr
0.41	0.53	0.49	0.51	0.017	0.021	13.2

**Table 2:** Main parameters of the used mills.

End Mill	Embossed Rating	Coating Chemical Composition	D, mm	ω, °	Number of Teeth, z
No. 1	UP210-S4-08020	AlCrSiN	8	35	4
No. 2	UP210-S6-12030	AlCrSiN	12	35	6

**Table 3:** Main parameters of the coating of the used mills.

Coating Chemical Composition	HV0.005, kgf/mm^2^	Friction Coefficient for Carbon Steel Dry Cutting, μ	Maximum Temperature, T, °C
AlCrSiN	3300	0.4	1100

**Table 4:** Milling machining conditions of the studied samples.

Mill	Sample	Milling Scheme	Milling Modes When the Mill Diameter is *d* = 8 mm: *s*_M_ (mm/min), *n* (rev/min), *B* (mm), *t* (mm)	Fx (*P_h_*) (N)	Fy (*P_v_*) (N)	Fxy (*P_hv_*) (N)
No. 1	Vertical	conventional	*S*_m_ = 14, *n* = 500, *B* = 2, *t* = 4	87.5	75	115.2
No. 1	Horizontal	conventional	*S*_m_ = 14, *n* = 500, *B* = 2, *t* = 4	89.5	72.5	115.2
No. 1	Vertical	conventional	*S*_m_ = 28, *n* = 500, *B* = 2, *t* = 4	130	122	178.3
No. 1	Horizontal	conventional	*S*_m_ = 28, *n* = 500, *B* = 2, *t* = 4	138	119	182.2
No. 2	Horizontal	climb	*S*_m_ = 56, *n* = 500, *B* = 2, *t* = 5.5	198	302	361.1
No. 2	Horizontal	climb	*S*_m_ = 56, *n* = 500, *B* = 2, *t* = 5.5	149	321	353.9
No. 2	Horizontal	climb	*S*_m_ = 84, *n* = 500, *B* = 2, *t* = 5.5	247	343	422.7
No. 2	Horizontal	climb	*S*_m_ = 84, *n* = 500, *B* = 2, *t* = 5.5	201	379	429

**Table 5:** Time ratio when milling the samples.

Feed (mm/min)	5.6	14	28	56	84
Time ratio	2.98	2.3	1.82	1.66	1.18

## Data Availability

The data presented in this study are available from the corresponding authors upon reasonable request.
